# Effects of medical resource capacities and intensities of public mitigation measures on outcomes of COVID-19 outbreaks

**DOI:** 10.1186/s12889-021-10657-4

**Published:** 2021-03-29

**Authors:** Xia Wang, Qian Li, Xiaodan Sun, Sha He, Fan Xia, Pengfei Song, Yiming Shao, Jianhong Wu, Robert A. Cheke, Sanyi Tang, Yanni Xiao

**Affiliations:** 1 School of Mathematics and Information Sciences, Shaanxi Normal University, Xi’an, 710119 P.R. China; 2 Department of Applied Mathematics, Xi’an Jiaotong University, Xi’an, 710049 P.R. China; 3 Chinese Center for Disease Control and Prevention, Beijing, 102206 P.R. China; 4 Laboratory for Industrial and Applied Mathematics, Department of Mathematics and Statistics, York University, Toronto, Ontario M3J 1P3 Canada; 5 Natural Resources Institute, University of Greenwich at Medway, Central Avenue, Chatham Maritime, Kent, ME4 4TB UK

**Keywords:** Pandemic, COVID-19, Model, Runs on medical resources, Inter-country comparisons, Prediction, Epidemiology

## Abstract

**Background:**

The COVID-19 pandemic is complex and is developing in different ways according to the country involved.

**Methods:**

To identify the key parameters or processes that have the greatest effects on the pandemic and reveal the different progressions of epidemics in different countries, we quantified enhanced control measures and the dynamics of the production and provision of medical resources. We then nested these within a COVID-19 epidemic transmission model, which is parameterized by multi-source data. We obtained rate functions related to the intensity of mitigation measures, the effective reproduction numbers and the timings and durations of runs on medical resources, given differing control measures implemented in various countries.

**Results:**

Increased detection rates may induce runs on medical resources and prolong their durations, depending on resource availability. Nevertheless, improving the detection rate can effectively and rapidly reduce the mortality rate, even after runs on medical resources. Combinations of multiple prevention and control strategies and timely improvement of abilities to supplement medical resources are key to effective control of the COVID-19 epidemic. A 50% reduction in comprehensive control measures would have led to the cumulative numbers of confirmed cases and deaths exceeding 590,000 and 60,000, respectively, by 27 March 2020 in mainland China.

**Conclusions:**

Multiple data sources and cross validation of a COVID-19 epidemic model, coupled with a medical resource logistic model, revealed the key factors that affect epidemic progressions and their outbreak patterns in different countries. These key factors are the type of emergency medical response to avoid runs on medical resources, especially improved detection rates, the ability to promote public health measures, and the synergistic effects of combinations of multiple prevention and control strategies. The proposed model can assist health authorities to predict when they will be most in need of hospital beds and equipment such as ventilators, personal protection equipment, drugs, and staff.

**Supplementary Information:**

The online version contains supplementary material available at 10.1186/s12889-021-10657-4.

## Background

In the absence of effective treatments for, or vaccines against, COVID-19, the early adoption of strict prevention and control measures will undoubtedly play a very important role in limiting the spread of the virus and the growth of the epidemic [1–8]. This has been shown by the successes of China, South Korea and other countries in curbing the spread of the virus and achieving important prevention and control results [7, 8]. For example, unprecedented restrictive measures, including travel restrictions, contact tracing, quarantine and lock-down of entire towns/cities adopted by the Chinese authorities has resulted in a significant reduction in the COVID-19 epidemic in mainland China [7, 8]. However, with the increase of COVID-19 cases in the world, especially the sharp increase in the cumulative number of reported deaths, the shortage of medical resources has become the most serious threat facing countries experiencing serious epidemics.

Enhancing the detection rate can lead to early quarantine or isolation of latent and/or infected individuals and then the numbers of confirmed cases increase significantly [2, 5, 8]. A common problem faced by many countries is to what extent can improvements to medical resources be synchronized with an increase of patient numbers to avoid runs on medical resources. How to quantify the levels of improvements in prevention and control measures and of medical resources to reveal differences in the epidemic’s progression in different countries remains unclear, but this question is addressed in this study. In order to investigate this issue, we propose a general COVID-19 epidemic transmission dynamics model that includes limitations of medical resources and enhancing prevention and control strategies in six selected countries (China, South Korea, Japan, Italy, Spain and Iran) [7, 8]. The formulated transmission model is parameterized by multi-source data such as the numbers of newly reported cases and the cumulative numbers of deaths for each country.

**Fig. 1:**
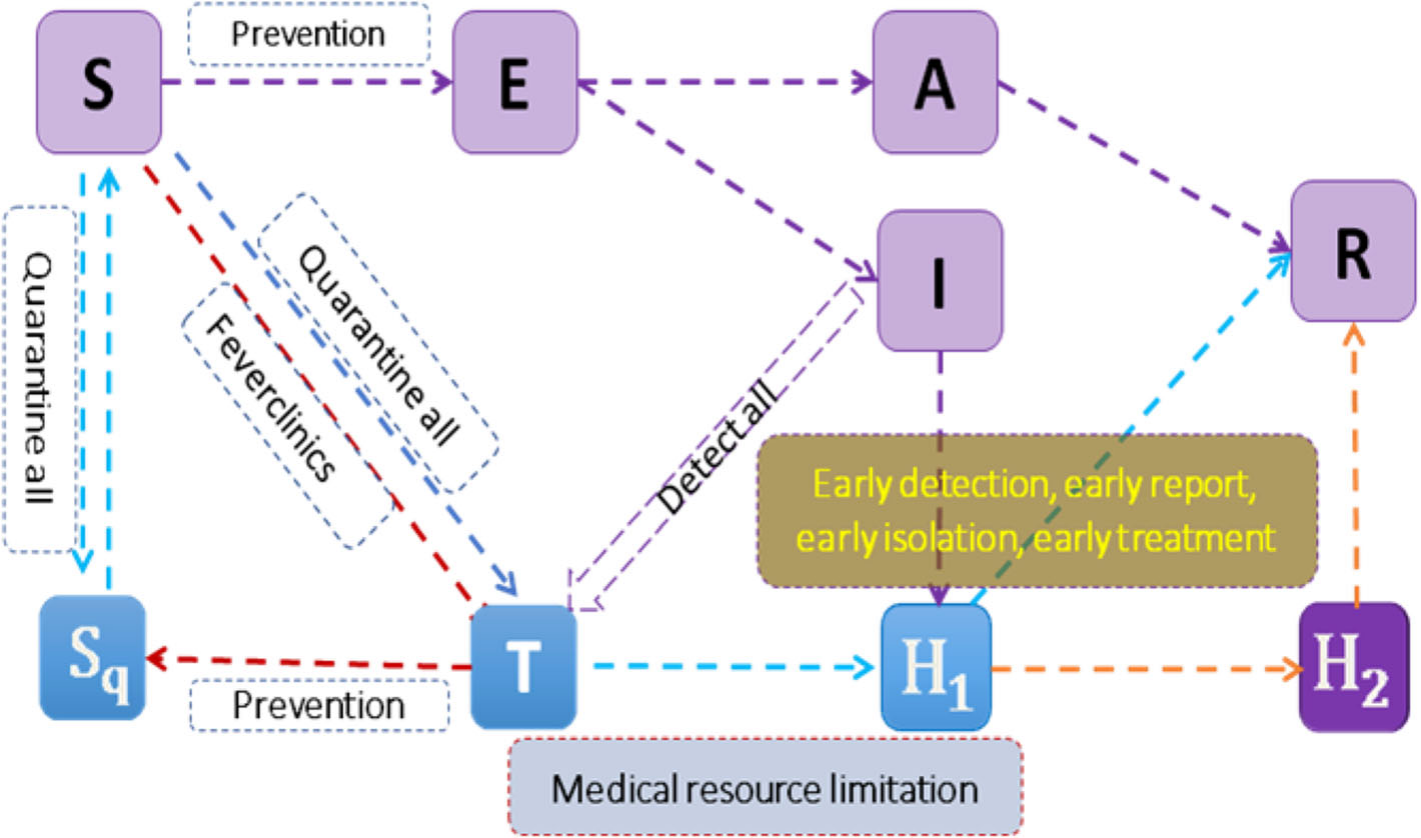
Flow diagram for the COVID-19 epidemic model incorporating containment and mitigation measures, where the medical resources limitation is described in terms of numbers of hospital beds

Using information such as the number of beds per thousand people in each country and differences in increasing volumes of medical resources (closely related to medical staff numbers) that can be provided by each country during public health emergencies [9–14], we modelled the number of beds provided by each country during the development of a COVID-19 epidemic with the logistic growth function using country-specific varying growth rates and carrying capacities (section SI1 of Supplementary information (SI)). In order to represent the limitation of hospital beds we divided confirmed cases into two groups in terms of severity of symptoms: non-hospitalized and hospitalized. The non-hospitalized individuals, who may later be admitted to hospital depending on the number of hospital beds available, can become a new source of infection, leading to new cases including family cluster infections. Hence the dynamics of hospital beds need to be nested within the transmission dynamics model to examine the level of improvement in medical resources on a COVID-19 epidemic in each country studied (Fig. 1 and Table 1).

**Table 1:** Parameter definitions for the COVID-19 epidemics

Parameter		Definitions
*c*(*t*)	*c*_0_	Contact rate at the initial time
	*c*_*b*_	Minimum contact rate under the current control strategies
	*r*_1_	Exponential decreasing rate of contact rate
*c*		Constant contact rate
*β*		Probability of infected individual’s transmission per contact
*q*(*t*)	*q*_0_	Quarantined rate of exposed individuals at the initial time
	*q*_*m*_	Maximum quarantined rate of exposed individuals under the current control strategies
	*r*_2_	Exponential increasing rate of quarantined rate of exposed individuals
*q*		Constant quarantined rate of exposed individuals
*ϑ*		Quarantined rate of susceptible individuals to the suspected class
*β*_*A*_		Probability of asymptomatic individual’s transmission per contact
*β*_*H*_		Probability of quarantined infected individual’s transmission per contact
*ρ*		Proportion of symptomatic infection
*σ*		Transition rate of exposed individuals to the infected class
*λ*		Rate at which the quarantined uninfected contacts were released into the wider community
*δ*_*I*_(*t*)	*δ*_*I*0_	Initial diagnosis rate of infected individuals
	*δ*_*If*_	Fastest diagnosis rate of infected individuals
	*r*_3_	Exponential increasing rate of diagnosis rate of infected individuals
*δ*_*I*_		Constant diagnosis rate of infected individuals
*δ*_*T*_(*t*)	*δ*_*T*0_	Initial diagnosis rate of quarantined individuals
	*δ*_*Tf*_	Fastest diagnosis rate of quarantined individuals
	*r*_4_	Exponential increasing rate of diagnosis rate of quarantined individuals
*δ*_*T*_		Constant diagnosis rate of quarantined individuals
*ω*		Confirmation ratio of quarantined exposed individuals
*θ*		Rate at which the confirmed infected individuals were hospitalized
*γ*_*A*_		Recovery rate of asymptomatic infected individuals
*γ*_1_		Recovery rate of confirmed infected individuals without hospitalization
*γ*_2_		Recovery rate of hospitalized infected individuals
*α*		Disease-induced death rate of infected individuals
*α*_1_		Disease-induced death rate of confirmed infected individuals without hospitalization
*α*_2_		Disease-induced death rate of hospitalized infected individuals
*H*_*c*_(*t*)	*H*_0_	The number of available hospital beds at the initial time
	*H*_*f*_	Maximum capacity of hospital beds
	*r*_*h*_	Exponential increasing rate of hospital beds
Initial values		Definitions
*S*(0)		Initial susceptible population
*E*(0)		Initial exposed population
*I*(0)		Initial infected population
*A*(0)		Initial asymptomatic population
*T*(0)		Initial suspected population
*S*_*q*_(0)		Initial quarantined susceptible population
*H*_1_(0)		Initial confirmed infected population without hospitalization
*H*_2_(0)		Initial hospitalized infected population
*R*(0)		Initial recovered population

## Methods

**Fig. 2:**
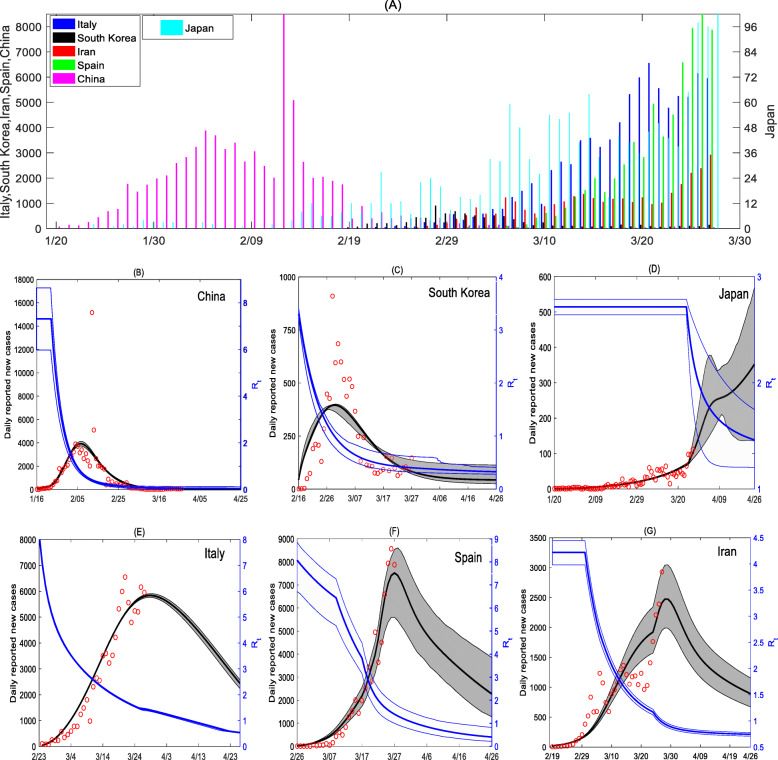
Data, curve fitting and effective reproduction numbers. **a**. Numbers of newly reported cases for China, South Korea, Japan, Iran, Italy, and Spain from 23 Jan to 27 March 2020. **b**–**g**. Numbers of newly reported cases (red open circles) for China (**b**), South Korea (**c**), Japan (**d**), Italy (**e**), Spain (**f**) and Iran (**g**) and data fitting and 95% confidence intervals (grey) with predictions for one more month. Blue lines are the effective reproduction numbers (*R*_*t*_) and their 95% confidence intervals

**Fig. 3:**
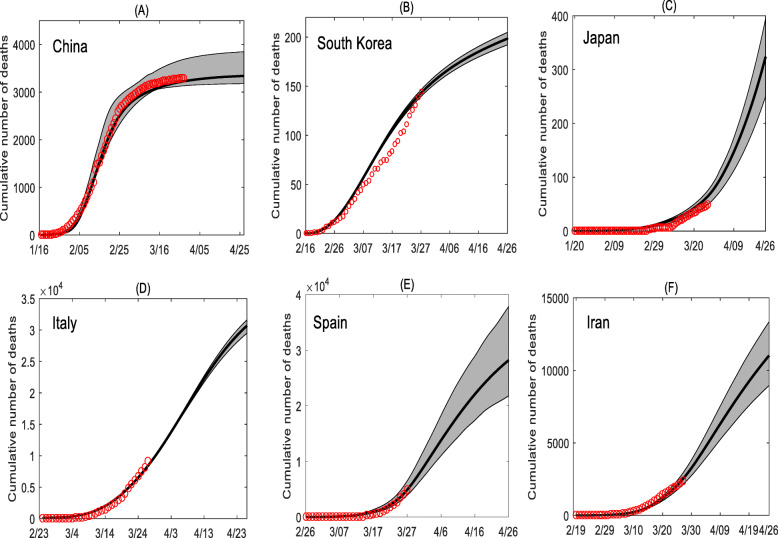
Data fitting and 95% confidence intervals of cumulative numbers of deaths for six countries indicated in each subplot

The multiple data sources including the numbers of newly reported cases and the cumulative numbers of reported deaths for all six countries were used to estimate the unknown parameters and to fit the data (Figs.2 and 3, Table 2). The parameter values associated with the intensity of disease transmission in each country are compared and discussed in SI3. As in China, the epidemic in South Korea became almost stable, and their respective effective reproduction numbers had, by mid-April 2020, both been less than 1 for six weeks (Fig. 2(b, c). The COVID-19 epidemic in Japan has been fluctuating on a small scale with a lot of random fluctuations. However, after 25 March the epidemic rebounded with the numbers of newly reported cases and deaths continuing to increase (Figs.2(d) and Fig. 3(c)). The Italian and Spanish epidemics seemed to be about to peak and they were approaching their turning points, but their cumulative death rates will continue to rise, and there is no sign of stabilization in the short term (Figs.2(e, f) and Fig. 3(d, e)). Finally, the epidemic in Iran has a strange trend, with repeated and huge fluctuations (Figs.2(g) and Fig. 3(f)).

**Table 2:** Parameter estimates for the COVID-19 epidemics in China, South Korea, Japan, Italy, Spain, and Iran

**Parameter**		**Estimated values**						**Source**
		**China**	**South Korea**	**Japan**	**Italy**	**Spain**	**Iran**	
*c*(*t*)	*c*_0_	12.2252	8.492	–	5.8547	5.0	4.995	Estimated
	*c*_*b*_	3.2332	3.6005	–	0.0105	0.4649	1.4678	Estimated
	*r*_1_	0.05	0.0237	–	0.0392	0.0532	0.101	Estimated
*c*		–	–	2.0	–	–	–	Estimated
*β*		0.1911	0.1911	0.1911	0.1911	0.1911	0.1701(Est)	(1, 2)
*q*(*t*)	*q*_0_	0.2128	0.1003	–	0.1007	0.1112	0.1995	Estimated
	*q*_*m*_	0.9	0.899	–	0.7106	0.6446	0.7963	Estimated
	*r*_2_	0.05	0.1206	–	0.1	0.046	0.0697	Estimated
*q*		–	–	0.8	–	–	–	Estimated
*ϑ*		1.0 × 10^−9^	0.0027	0.01	1.4561 × 10^−9^	4.682 × 10^−6^	4.98 × 10^−9^	Estimated
*β*_*A*_		0.001	0.0484	0.1028	0.15	0.1227	0.171	Estimated
*β*_*H*_		0.001	0.0043	1.0051 × 10^−4^	0.15	0.053	0.013	Estimated
*ρ*		0.45	0.95	0.4	0.4575	0.5556	0.6(7)	Estimated
*σ*		1/5	1/5	1/5	1/5	1/5	1/5	(8)
*λ*		1/14	1/14	1/14	1/14	1/14	1/14	(1)
*δ*_*I*_(*t*)	*δ*_*I*0_	0.15	–	0.02*	0.099	0.0512	0.1447	Estimated
	*δ*_*If*_	0.8071	–	0.4988	0.2001	0.1725	0.3337	Estimated
	*r*_3_	0.1	–	0.2999	0.3495	0.3401	0.2998	Estimated
*δ*_*I*_		–	0.4308	–	–	–	–	Estimated
*δ*_*T*_(*t*)	*δ*_*T*0_	–	–	0.05*	0.0979	0.1093	0.1431	Estimated
	*δ*_*Tf*_	–	–	0.4509	0.3329	0.4245	0.334	Estimated
	*r*_4_	–	–	0.103	0.3995	0.3317	0.1	Estimated
*δ*_*T*_		0.5	0.3835	–	–	–	–	Estimated
*ω*		0.1001	0.0089	0.2912	0.7999	0.7845	0.4931	Estimated
*θ*		0.4892	0.336	0.7732	0.3333	0.1677	0.3014	Estimated
*γ*_*A*_		0.18	0.1807	0.1	0.1468	0.1442	0.2014	Estimated
*γ*_1_		0.04	0.1359	0.0989	0.08	0.085	0.0498	Estimated
*γ*_2_		0.08	0.0926	0.0667	0.0857	0.0903	0.0667	Estimated
*α*		0.01	0.005	1.0008 × 10^−4^	3.8099 × 10^−4^	0.0231	0.0212	Estimated
*α*_1_		0.01	0.002	1.0066 × 10^−4^	0.0284	0.0201	0.012	Estimated
*α*_2_		7.6724 × 10^−4^	0.0024	0.0053	0.01	0.0134	0.01	Estimated
*H*_*c*_(*t*)	*H*_0_	1500	796.679	–	1997.7	1200	999.1962	Estimated
	*H*_*f*_	4.1968 × 10^4^	3. 0065 × 10^3^	–	3.6 × 10^4^	3.0 ×10^4^	1.6235 ×10^4^	Estimated
	*r*_*h*_	0.15	0.9728	–	0.1650	0.077	0.05	Estimated
**Initial values**		**Estimated values**						**Source**
		**China**	**South Korea**	**Japan**	**Italy**	**Spain**	**Iran**	
*S*(0)		1.0 × 10^8^	2.0651 × 10^6^	2.9985 × 10^6^	2.0 × 10^6^	2.0 × 10^6^	3.9945 × 10^6^	Estimated
*E*(0)		1000	999.919	10.0	2435.6	2500	149.2259	Estimated
*I*(0)		400	99.6953	10.0	275.9334	400	209.0317	Estimated
*A*(0)		414.5036	199.6778	79.9999	250.3936	280	140.2632	Estimated
*T*(0)		2000	105.078	5.0	0.0001	0	0 (Assumed)	Estimated
*S*_*q*_(0)		2000	7123 (Data)	307.594	4.2 × 10^−5^	7.4	0 (Assumed)	Estimated
*H*_1_(0)		0	0	0	22	10	0	Data
*H*_2_(0)		28	21	0	80	10	0	Data
*R*(0)		15	9	1	1	0	0	Data

Note that, ‘Est’ means that the parameter values are estimated by fitting the models to the data when the source column indicates that they are not. ‘*’ means the estimated values for *δ*_*I*_ and *δ*_*T*_ before 2020/3/25, otherwise *δ*_*I*_ and *δ*_*T*_ are increasing functions with respect to time *t*

**Fig. 4:**
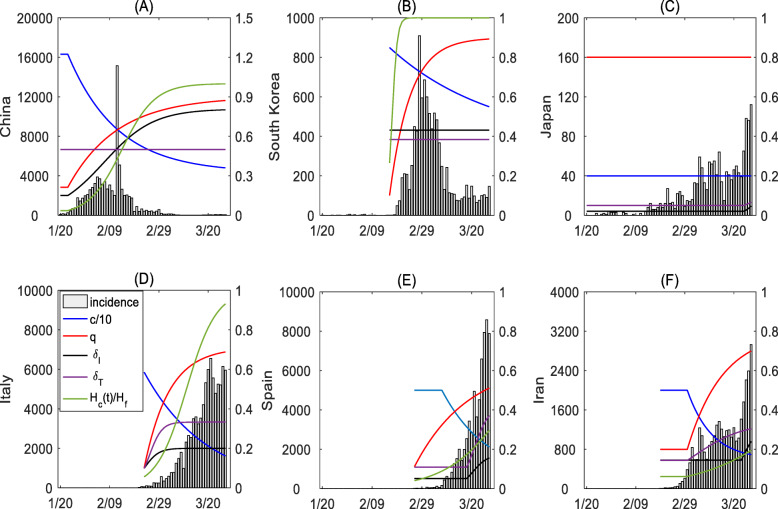
The synergistic effects of comprehensive intervention strategies and capacities of medical resources. Numbers of newly reported cases for China (**a**), South Korea (**b**), Japan (**c**), Italy (**d**), Spain (**e**) and Iran (**f**) from 23 Jan to 27 March 2020, and improving containment and mitigation measures including functions for contact rate (blue lines), quarantine rate (red), detection rate *δ*_*I*_ (black), detection rate *δ*_*T*_ (purple) and medical resources *H*_*c*_ (number of beds for each country, green). The contact rates have been divided by 10, and the numbers of beds have been divided by the carrying capacity *H*_*f*_ in each subplot

In order to reveal the complex patterns and huge differences in the COVID-19 epidemics among the various countries shown in Figs.2 and 3, we compared the effectiveness and timeliness of the continuously strengthened comprehensive prevention and control strategies in various countries (Fig. 4), with a view to increasing our understanding and making suggestions for future prevention and control strategies. To do this, we quantified the intensities of the control measures against COVID-19 epidemics for each country by estimating the evolution of the contact rate (*c(t)*), quarantine rate (*q(t)*), detection rates (*δ*_*I*_(t) and δ_*T*_(*t*)) and medical resource capacity (*H*_*c*_(*t*)) (see sections SI2 and SI3).

**Fig. 5:**
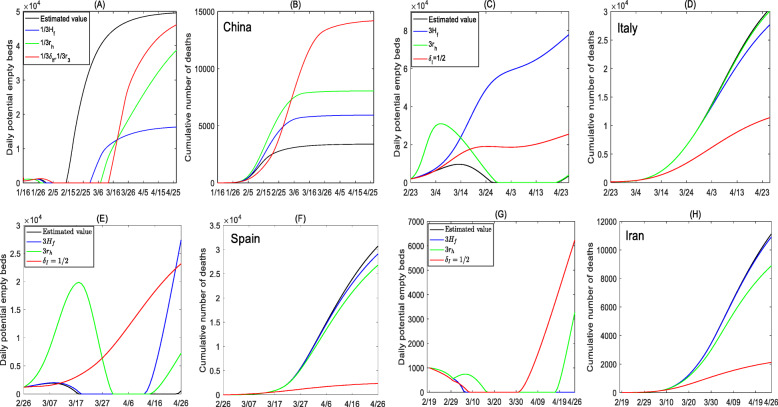
Sensitivity analyses reveal the relation between daily numbers of potential empty beds and cumulative numbers of deaths. The effects of the production capacity and reserve capacities of medical resources, and detection rates on runs on medical resources and cumulative numbers of deaths for China (**a** & **b**), Italy (**c** & **d**), Spain (**e** & **f**) and Iran (**g** & **h**). The daily potential numbers of empty beds were calculated from the formula max {*H*_*c*_(*t*) − *H*_2_(*t*) − *θH*_1_(*t*), 0}. The black curves in each subplot are generated by the baseline parameter values listed in Table 2. The values (Table 3) of daily potential numbers of empty beds and cumulative numbers of deaths on 26 April 2020 have been calculated to reveal when and how long the runs on medical resources could last, depending on the production capacities of medical resources and detection rates for each country, when each parameter value is reduced by 1/3

Define *H*_*r*_(*t*) = max {*H*_*c*_(*t*) − *H*_2_(*t*) − *θH*_1_(*t*), 0} as the daily potential number of beds available for the individuals currently quarantined at home . Then *H*_*r*_(t) = 0 for certain days means the occurrence of a run on medical resources in some countries. In order to identify the timing of such runs for various countries, reveal the variation in the cumulative number of deaths with the detection rate, maximum number of beds and production capacity of beds, we numerically calculated *H*_*r*_(t) and the cumulative number of deaths (Fig. 5 and Table 3).

**Table 3:** The effects of the production capacities and reserves of medical resources, and detection rates on runs on medical resources and cumulative numbers of deaths on 26 April 2020

Value of parameter	Daily potential empty beds (DPEB)			The cumulative number of death cases (CNDC)			Value of parameter	DPEB	CNDC
	Italy	Spain	Iran	Italy	Spain	Iran		China	
***Estimated*** ***value***	3539	545	0	30,509	30,709	11,118	***Estimated*** ***value***	49,598	3397
**3*H***_***f***_	77,829	27,400	0	27,684 (−9.3%)	29,099 (−5.2%)	10,903 (−1.9%)	**1**/**3*H***_***f***_	16,284 (−67.17%)	5933 (74.6%)
**3*r***_***h***_	3896	7254	3264	30,103 (−1.3%)	26,793 (−12.8%)	8890 (−20%)	**1**/**3*r***_***h***_	38,705 (−21.96%)	8053 (137%)
***δ***_***I***_ ***=*** **1**/**2**	25,487	23,170	6322	11,399 (−62.6%)	2350 (−92.3%)	2122 (−80.9%)	**1**/**3*δ***_***If***_ **1**/**3*r***_**3**_	46,109 (−7.0%)	14,187 (317.6%)

## Results

It follows from Fig. 4 that five of the six countries, but not Japan, were constantly increasing their numbers of beds available (*H*_*c*_(*t*), green curves) for COVID-19 patients with the development of the epidemic in accordance with each country’s medical capacity. The rates of increases in the numbers of beds for countries other than Japan are in the order South Korea, China, Italy, Spain and Iran. South Korea and China would soon be able to reach the maximum number of medical beds needed after their control intensity improvements (Fig. 4(a) and (b)). Due to the low rate of increase of infected individuals in Japan, so far there is no urgent need to supplement the number of beds for high-risk patients there (shown in Fig. 4(c)). The contact rate function (blue curve in Fig. 4) in China has declined very fast since 23 January when Wuhan city and all parts of the country continued to take stringent control measures. The response speed of increasing social distancing and strengthening self-quarantine measures was very fast in mainland China, while South Korea’s, Italy’s and Spain’s contact rate functions gradually decreased. Italy and Spain have relatively low quarantine rates (red curves in Fig. 4) while the other four countries all have high contact tracing followed by quarantine. Japan’s quarantine rate remains at a high level so far. The relatively high detection rates, followed by strict quarantine, are associated with quick control of the epidemic (as illustrated for China, South Korea and Iran in Fig. 4(a, b, f)) while the epidemics in Italy and Spain with low detection rates became worse (Fig. 4(d, e)).

By calculating the daily potential number of beds available for the individuals currently quarantined at home, *H*_*r*_(*t*), with the estimated parameters, we see that the short-term shortage of medical resources in February occurred in mainland China from 1 to 13 February. However, after the specially built Huoshenshan Hospital began to treat patients on 4 February and *Fangcang* (shelter/observation ward) hospitals began to treat patients on 5 February, the number of beds increased rapidly, effectively alleviating the shortage of medical resources. However, the cumulative number of deaths would increase by 74.6% (or 137%), if the capacity (or growth rate) of beds were reduced from *H*_*f*_ to 1/3*H*_*f*_ (or *r*_3_ to 1/3*r*_3_) (Fig. 5 (a-b) and Table 3). If the two parameters δ_If_ ( related to the detection ability) and *r*_3_ were reduced at the same time to 1/3 of the estimated values, then the duration of the run on medical resources would increase significantly (from 12 to 37 days) and consequently the cumulative number of deaths would increase rapidly, up to 317.6% by 26 April (Fig. 5 (a-b)). If the detection rate is low, the run on resources will occur later, but eventually it will occur in a wider time range, which will lead to more people becoming infected and more deaths. This indicates that rapidly increased supplies of medical resources and disease detection effectively reduced the mortality in China.

Increasing the provision of beds (3*r*_*h*_here) threefold in Italy, Spain and Iran will only alleviate the shortage of resources for a short time, and then the run on medical resources will happen again soon afterwards (green curves in Fig. 5(c,e,g)). Italy and Spain began to recover slowly, after almost 25 days and 15 days, respectively, with zero beds remaining. Iran began to recover at a faster speed after a long period with zero beds remaining. Nevertheless, Iran had the largest reduction in the number of cumulative deaths (a reduction of 20%), followed by Spain (12.8% reduction) (Table 3). If the maximum number of beds is only increased to 3*H*_*f*_, the results shown in Fig. 5 clarify that there is little impact on the death toll in Spain and Iran (Fig. 5(f, h)). Increases in the detection rate in Italy, Spain and Iran greatly reduces the cumulative number of deaths (red curves in Fig. 5(d, f, h). This is because increasing the detection rate leads to significant declines in the numbers of new infections, due to strict quarantine and isolation strategies, which not only decreases the number of deaths but also avoids runs on medical resources (red curves in Fig. 5(c,e)). Furthermore, in Iran increasing the detection rate still induces a run on resources in the early stage of the epidemic but leads to recovery at a later stage due to reduced numbers of new infections (black curves in Fig. 5(e)), implicating the weak medical resources in Iran.

Having taken continuous and strengthened prevention and control measures, China and South Korea have quickly controlled the epidemic. The key processes for quick mitigation of the epidemic are the intensity of prevention and control measures represented by the four rates *r*_1_*, r*_2_*, r*_3_ and *r*_*h*_: contact rate declines, quarantine rate increases, decreasing periods for detection and increasing rates of medical resource production, respectively (see SI for explanations). Reducing these rates by 10, 20, 30, 40 and 50% simultaneously would have resulted in increased fractions in the cumulative numbers of confirmed cases and deaths (Table 4). Comparing the changing rates indicates that China’s control efficacy is better than South Korea’s, especially in terms of reducing deaths. Given a reduction by 50% in the comprehensive control measures in China, the cumulative number of confirmed cases and deaths would have exceeded 590,000 and 60,000, respectively, by 27 March. Therefore, without the very strong and comprehensive prevention and control measures that were invoked [7, 8], the development of China’s epidemic would have been unimaginable, with exceedingly large numbers of cases and a surprisingly high death toll.

**Table 4:** The effects of decreasing *r*_1_*, r*_2_*, r*_3_ and *r*_*h*_ on the cumulative numbers of confirmed cases and cumulative numbers of deaths on 27 March 2020 in China and South Korea

Cases	The cumulative number of confirmed cases		The cumulative number of deaths	
	China	South Korea	China	South Korea
Case *C*_1_	8.256×10^4^	9477	3329	170
Case *C*_2_	1.0426×10^5^(+ 26.3%)	10,693 (+ 12.8%)	5175 (+ 55.4%)	190 (+ 11.8%)
Case *C*_3_	1.3881×10^5^(+ 68.1%)	12,401 (+ 30.9%)	8618 (+ 158.8%)	217 (27.6%)
Case *C*_4_	1.9892×10^5^(+ 141.0%)	14,941 (+ 57.7%)	15,396 (+ 362.4%)	251 (+ 47.6%)
Case *C*_5_	3.1711×10^5^(+ 284.1%)	18,962 (+ 100.0%)	30,027 (+ 801.9%)	297 (+ 74.7%)
Case *C*_6_	5.9333×10^5^(+ 618.7%)	25,864 (+ 172.9%)	63,657 (+ 1811.9%)	370 (+ 117.6%)

Note that *r*_1_, *r*_2_, *r*_3_, and *r*_*h*_ are the estimated values for corresponding countries, and for South Korea *r*_3_ = 0 due to its constant diagnosis rate in Case *C*_1_. For Cases *C*_2_, *C*_3_, *C*_4_, *C*_5_ and *C*_6_, the four rates *r*_1_, *r*_2_, *r*_3_, and *r*_*h*_ were simultaneously reduced by 10, 20, 30, 40 and 50%, respectively

## Discussion

It is known that the marked differences in the epidemics in various countries are associated with differences in the implementation of multiple strategies for public interventions together with increasing medical resource capacities. By estimating time-dependent contact, quarantine and detection rates, we quantified enhancements to prevention and control strategies, and modelled the dynamics of medical resources, which were nested within the epidemic model proposed, revealing the timings of runs on medical resources in six different countries. Thus, the model could be used to assist health authorities to predict when they will be most in need of hospital beds, equipment such as ventilators, personal protection equipment (PPE), drugs and medical staff. The model also describes the effect of improving control measures on the complex patterns of epidemics in different countries, shows that detection rates are crucial for reducing morbidity and mortality and that synergies between prevention and control measures and medical resource availability are essential for successful control of COVID-19.

Detection of infections is a key process that significantly affects the numbers of confirmed cases and deaths. This is especially so for an increasing detection rate, which, while increasing the number of confirmed cases in the short term, leads to declines in the number of new infections. This is due to strict contact tracing followed by quarantine and isolation, and consequently reductions in the number of confirmed cases and deaths in the long term. Moreover, increasing the detection rate may result in runs on medical resources, depending on their initial capacities. As illustrated in Fig. 5 (C, E and G) merely increasing detection rates in Italy or Spain did not induce runs on medical resources but these did occur in Iran (red curves). Hence the synergistic effect of improving medical capacity and production with enhancing detection is essential to mitigating the COVID-19 pandemic as well as avoiding runs on medical resources.

We suggest that Japan should pay more attention to increasing medical resources as its detection rate has increased since 25 March, otherwise the numbers of confirmed cases and deaths will increase quickly as the intensity of its control measures is not as high as in South Korea or even in Iran. If Iran had medical conditions equivalent to those of South Korea, the effects of its control measures would be far more effective in controlling the epidemic and the current situation would be less severe. Comparing the estimated rate functions for Italy, Spain and Iran indicates that a low detection rate is a key process that significantly affects the epidemic, while Iran is mainly affected by its limited medical resources. Regardless of other factors, improving the detection rate can effectively and rapidly reduce the mortality rate, even after runs on medical resources. Therefore, in order to effectively reduce the numbers of new infections and mortality in COVID-19 outbreaks, detection rates should be increased while improving the production and capacity of medical resources. The synergistic effects of comprehensive prevention and control strategies are likely to succeed in mitigating epidemics, as shown by the experience of China and South Korea [1–3, 5–8], from whose examples other countries can learn.

We also recognize that there are some limitations to our modelling practice. The proposed model contains many parameters, which are not identifiable based on the observed epidemic data only. It may be necessary to conduct parameter identifiability analysis or use more of the literature to determine the values for some of these parameters more accurately. Currently, the COVID-19 epidemic is still very severe, but the development and deployment of vaccines provides optimism that the pandemic can be controlled. Much recent research has focused on the effect of vaccination on COVID-19 epidemics [15–17] and the design of optimal vaccination strategies [18, 19]. So, considering the limited medical resources, future research should focus on questions such as how to use the vaccine resources effectively, to what extent can vaccination alleviate the problem of insufficient medical resources, and how should control strategies change after vaccinations?

## Conclusions

The key factors that affect epidemic progressions and their outbreak patterns in different countries are the types of emergency medical response to avoid runs on medical resources. While increasing the detection rate can effectively reduce the mortality rate and the number of confirmed cases, it may result in increasing numbers of confirmed cases in short term and then runs on medical resources. More attention should be paid to the synergistic effect of improving medical capacity and production with enhancing detection, to mitigate the COVID-19 pandemic as well as avoiding runs on medical resources. The proposed model can assist health authorities to predict when they will be most in need of hospital beds and equipment such as ventilators, personal protection equipment, drugs, and staff.

## Supplementary Information

**Additional file 1: SI1.** Model formulation. **SI2.** The definition of rate functions. **SI3.** Comparison of the intensity of control measures based on parameter values. **SI4.** The data and uncertainty analysis.

## Data Availability

The data generating the findings of this article are included within the article and websites shown in the supplementary information (SI4). The public access to the databases cited in the supplementary material is open.

## Abbreviations

COVID-19: Coronavirus Disease 2019
PPE: Personal Protection Equipment
