# Supplementary material for: Effects of medical resource capacities and intensities of public mitigation measures on outcomes of COVID-19 outbreaks
Source: BMC Public Health. 2021 Mar 29;21:605. doi: 10.1186/s12889-021-10657-4 (PMC8006107; doi:10.1186/s12889-021-10657-4)
Supplement: Supplementary file 1 — Additional file 1: SI1. Model formulation. SI2. The definition of rate functions. SI3. Comparison of the intensity of control measures based on parameter values. SI4. The data and uncertainty analysis. [file 12889_2021_10657_MOESM1_ESM.docx]

**Supplementary information (SI)**

Effects of medical resource capacities and intensities of public mitigation measures on outcomes of COVID-19 outbreaks

This supplementary information (SI) provides a detailed model description, definitions of all of the rate functions, and comparisons of the intensities of control measures based on identified parameter values. In addition, medical resource limitations for each country are described and discussed in terms of numbers of hospital beds.

**SI1. The transmission model incorporating interventions**

In order to reveal the marked differences between the applications of various control strategies independently or simultaneously under medical resource limitation, we propose a generalized COVID-19 epidemic transmission dynamics model as shown in Fig.1 [1, 2]:

$$\left\{ \begin{aligned} S^{'}=-\left( c\left( t \right)\beta+q\left( t \right)c\left( t \right)\left( 1-\beta\right) \right)SI/N-{S(\beta}_{A}c\left( t \right)A+\beta_{H}H_{1})/N-\vartheta S+\lambda S_{q}, \\ E^{'}=\left( 1-q\left( t \right) \right)c\left( t \right)\beta SI/N+{S(\beta}_{A}c\left( t \right)A+\beta_{H}H_{1})/N-\sigma E, \\ I^{'} = \sigma\rho E-\alpha I-\delta_{I}\left( t \right)I, \\ A^{'}= \sigma\left( 1-\rho\right)E-\gamma_{A} A, \\ S_{q}^{'}=q\left( t \right)c\left( t \right)\left( 1-\beta\right)SI/N+\left( 1-\omega\right)\delta_{T}\left( t \right)T-\lambda S_{q}, (1) \\ T^{'}=q\left( t \right)c\left( t \right)\beta SI/N+\vartheta S-\delta_{T}\left( t \right)T, \\ H_{1}^{'}=\delta_{I}\left( t \right)I+\omega\delta_{T}\left( t \right)T-\min\left\{ \theta H_{1},\max\left\{ \left( H_{c}\left( t \right)-H_{2} \right),0 \right\} \right\}-\gamma_{1}H_{1}-\alpha_{1}H_{1}, \\ H_{2}^{'}=\min\left\{ \theta H_{1},\max\left\{ \left( H_{c}\left( t \right)-H_{2} \right),0 \right\} \right\}-\gamma_{2}H_{2}-\alpha_{2}H_{2}, \\ R^{'}={\gamma_{A} A+\gamma}_{1}H_{1}+\gamma_{2}H_{2}. \end{aligned} \right.$$

In this model, $S,S_{q},E,T,I,{A, H}_{1},H_{2},R$ represent the populations of susceptible ($S$), quarantined susceptible with contact tracing ($S_{q}$), exposed ($E$), quarantined suspected with contact tracing ($T$) including patients visiting fever clinics, infected ($I$) with symptoms, infected ($A$) but asymptomatic, people after a first medical visit but without a confirmed COVID-19 diagnosis and who are requested to quarantine at home ($H_{1}$), hospitalized and confirmed ($H_{2}$) and recovered ($R$).$N$ denotes the total population, and $H_{c}\left( t \right)$represents the capacity of hospital beds at time t which is used to describe the medical resource capacity. The detailed definitions can be found in section SI2.

In model (1), through contact tracing a proportion, $q$, of individuals exposed to the virus is quarantined, and can either move to compartment $T$ $E_{q}$ or $S_{q}$, depending on whether they are infected or not. The other proportion, 1 – q$1-q$, consists of individuals exposed to the virus who are missed from contact tracing and move to the exposed compartment $E$when they become infectious, or else they stay in compartment $S$. Further, we denote the transmission probability by β and the contact rate by c. Then, the quarantined individuals, if infected (or uninfected), move to compartment $T$ (or$S_{q}$ $S_{q}$) at a rate of $\beta cq$ $\beta cq$ (or $(1 - \beta)cq)$). Those who are not quarantined, if infected, will move to compartment $E$ at a rate of$\beta c(1-q)$. We denote by constant $\vartheta$ the transition rate from the susceptible class to the suspected compartment via general clinical medication due to fever or other symptoms. Meanwhile, the transmissibility of asymptomatic patients is lower than that of symptomatic patients, thus$\beta_{A}<\beta$. Further, those confirmed but not hospitalized can transmit virus to their family members, or others, during their visits to health care facilities and grocery stores etc. so $\beta_{H}<\beta$.

We note that the data on suspected individuals, and also most of the confirmed cases, come from the compartment $T$ in China and South Korea. The suspected individuals leave this compartment at a rate $\delta_{T}\left( t \right)$, with a proportion,$\omega$*,* confirmed to be infected by COVID-19 going to the compartment$H_{1}$, whilst the other proportion, 1-$\omega$*,* proven to be uninfected moves to the quarantined susceptible class$S_{q}$ once recovered. People confirmed with COVID-19 may not be hospitalized due to a limitation of hospital beds, thus the term $min\{ \theta H_{1}, max\{\left( H_{c}(t)-H_{2} \right),0\}\}$ is used to describe the maximum number of newly hospitalized per day to maximize hospital utilization, which is a piecewise function according to the relationship between $\theta H_{1}$ and $max\{\left( H_{c}(t)-H_{2} \right),0\}$, where$\theta H_{1}$is the number of confirmed cases who are waiting for beds on day *t*. $H_{c}(t)$ is the number of hospital beds at time *t.* Therefore, $\max\left\{ \left( H_{c}\left( t \right)-H_{2} \right),0 \right\}$is the maximum number of remaining beds on the day. The detailed definitions of all parameters are given in Table S1.

**SI2. Definition of rate functions**

As governments in various countries have been gradually strengthening their protection measures and medical resources and, in particular, providing more and more beds in response to the epidemic, we model the evolving number of beds by the logistic growth model

$$\frac{dH_{c}(t)}{dt}=r_{h}H_{c}\left( t \right)\left[ 1-\frac{H_{c}\left( t \right)}{H_{f}} \right],$$

where$r_{h}$ indicates the country-specific production capacity of medical resources in response to emerging infectious diseases and $H_{f}$ denotes the country-specific maximum number of beds that can be provided during the disease outbreak. Therefore, these two parameters reflect the capacity of medical resources of each country in response to COVID-19 outbreaks. In the early stage of an outbreak, due to adequate quantities of medical resources or insufficient understanding of the hospital bed needs, the number of beds is basically constant. Therefore, solving the above logistic equation, we have the number of beds on each day according to the following piecewise function:

$$H_{c}\left( t \right)=\left\{ \begin{aligned} H_{0}, t\leq T_{c}, \\ \frac{H_{0}H_{f}}{H_{0}+(H_{f}-H_{0})e^{-r_{h}(t-T_{c})}}, t>T_{c,} \end{aligned} \right.$$

where $H_{0}$indicates the number of initial beds that can be provided to patients with COVID-19 at the beginning of the outbreak, and $T_{c}$ denotes the critical time when each country starts to increase medical resources including hospital beds. Such critical times were 23 January and 1 March 2020 for China and Iran, respectively, while $T_{c}$ was zero for Italy, Spain and South Korea, i.e. at the beginning of the epidemic the medical resources of these countries were constantly being supplemented. In particular, before 25 March, due to the small number of confirmed cases, Japan was not limited by any medical resources, so it is assumed that all confirmed cases were hospitalized in time or isolated at home.

Since different containment and mitigation strategies have been implemented in different countries with different critical times and intensities of strengthening prevention and control measures [3-9], the functions related to contact, detection and quarantine rates in the model should be defined as piecewise functions, and the corresponding parameter values can be used to reflect the intensity at which control measures are implemented in each country. We define $c\left( t \right)$, $q(t)$, $\delta_{I}(t)$ and $\delta_{T}(t)$ below. Note that the two functions $c\left( t \right)$ and $q(t)$ are fixed as constants for Japan during the outbreaks, and the critical time for all rate functions defined in the following is 23 January for China, at the beginning of the epidemic for Italy.

In order to accurately describe the gradual strengthening of control strategies in this model, we assume that with increasing intensity of a control strategy the contact rate $c\left( t \right)$ is a decreasing function with respect to time *t*, given by [1,2]

$$c\left( t \right)=\left\{ \begin{aligned} c_{0}, t\leq T_{c}, \\ {(c}_{0}-c_{b})e^{-r_{1}\left( t-T_{c} \right)}+c_{b}, t>T_{c}, \end{aligned} \right.$$

where$c_{0}$ denotes the baseline contact rate at the initial time with $c\left( 0 \right)=c_{0}$, $c_{b}$ denotes the minimum contact rate under the current control strategies with $\lim_{t\to\infty} c\left( t \right)=c_{b}$, where $c_{b}<c_{0}$, and $r_{1}$ denotes how an exponential decrease in the contact rate is achieved. The critical time for Spain is 7 March [5], and the critical times for other countries are the same as those in function $H_{c}\left( t \right)$.

Similarly, to characterize enhanced contact tracing we define $q\left( t \right)$as an increasing function with respect to time *t*, written as

$$q\left( t \right)=\left\{ \begin{aligned} q_{0}, t\leq T_{c}, \\ \left( q_{0}-q_{m} \right)e^{{-r}_{2}(t-T_{c})}+q_{m}, t>T_{c}, \end{aligned} \right.$$

where $q_{0}$ is the initial quarantined rate of exposed individuals with $q\left( 0 \right)=q_{0}$ for China, South Korea, Italy, Spain and Iran, $q_{m}$ is the maximum quarantined rate under the current control strategies with $\lim_{t\to\infty} q\left( t \right)=q_{m}$ and $q_{m}>q_{0}$, and$r_{2}$ represents how an exponential increase in the quarantined rate is achieved. The critical times for each country are the same as those in function $H_{c}\left( t \right)$.

We also set the detection rate $\delta_{I}(t)$ as an increasing function with respect to time *t*, thus the detection period $1/\delta_{I}\left( t \right)$ is a decreasing function of *t* with the following form:

$$\frac{1}{\delta_{I}\left( t \right)}=\left\{ \begin{aligned} \frac{1}{\delta_{I0}}, t\leq T_{c}, \\ \left( \frac{1}{\delta_{I0}}-\frac{1}{\delta_{If}} \right)e^{-r_{3}(t-T_{c})}+\frac{1}{\delta_{If}}, t>T_{c}, \end{aligned} \right.$$

where $\delta_{I0}$ is the initial rate of confirmation, $\delta_{If}$ is the fastest confirmation rate, and $r_{3}$ is the exponentially decreasing rate of the detection period. We define $\delta_{I}\left( 0 \right)=\delta_{I0}$ and $\lim_{t\to\infty} \delta_{I}\left( t \right)=\delta_{If}$ with $\delta_{If}>\delta_{I0}$. $\frac{1}{\delta_{T}\left( t \right)}$can be similarly defined. Note that both $\delta_{I}(t)$ and $\delta_{T}(t)$ are constants for South Korea, indicating that South Korea had a high detection rate from the beginning of the epidemic. Due to the development of the epidemic, Japan began to gradually improve its detection rate from 25 March, so the critical time for both of these detection rate functions is 25 March. The critical time for both detection rate functions $\delta_{I}\left( t \right)$ and $\delta_{T}(t)$ is 17 March in Spain [5]. Similarly, due to its limited medical resources, Iran first strengthened the detection rate of suspected cases on 1 March [4], and then further expanded the detection range on 23 March, therefore the critical time for $\delta_{T}(t)$ is 1 March, and for $\delta_{I}\left( t \right)$it is 23 March.

**SI3. Comparison of the intensity of control measures based on parameter values**

**Detection rates**$\delta_{I}\mathrm{and}\delta_{I}(t)$, $\delta_{T}\mathrm{and}\delta_{T}(t)$

As the definition of the number of confirmed cases from the population waiting to be tested (i.e. the $T$ class) has not considered the process of passing the incubation period, the baseline parameter values of $\delta_{I}(t)$ in some countries are greater than those of $\delta_{T}(t)$, as shown in Table S2. The detection rates of Japan, Italy and Spain are relatively lower than those for China and South Korea. However, Japan did not gradually improve its detection rate until 25 March, while the detection rate for Iran was increasing and finally tended to a level almost similar to that for China, which indicates that Iran is constantly improving its detection rate.

**Quarantine intensity**$q \mathrm{or} q(t)$

The isolation rate$q$ in Japan has been very high since the outbreak, which fully reflects the high intensity of self-isolation in Japan, with the strong self-discipline of its citizens being one of the important factors resulting in the low level of the outbreak. The final quarantine rates of China, South Korea and Iran tend to be close to those of Japan with a relatively high growth rate $r_{2}$. In contrast, the baseline values of quarantine rates for Italy and Spain are relative low, and the growth rate for Spain is smaller than those of all other countries.

**Contact rate**$c \mathrm{or} c(t)$

There is no doubt that early in the outbreak in China, the number of contacts with susceptible persons per infected person was the largest. The final contact numbers of Italy, Spain and Iran are relatively low, which indicates that these countries had escalated social distancing measures in the later period. If they continue to maintain this level of measures for a long enough period, the outbreak can be halted. However, Japan has always adopted a relatively mild prevention and control strategy, and its exposure number has been maintained at a high level, while the limit value$c_{b}$ of South Korea is relatively large. This reveals that although the cumulative number of reported cases in these two countries is not large at present, the number of newly reported cases may increase repeatedly in the near future. This could be particularly serious for Japan.

**Capacity of medical resources** $H_{c}\mathrm{and}H_{c}(t)$

Due to the low cumulative number of confirmed cases and the low number of newly reported cases, there is no problem of limited medical resources in Japan so far, but with the development of the epidemic, whether there will be a run on medical resources or not remains to be seen. The production capacity$r_{h}$ of medical resources in Italy is the largest except for South Korea, and then China. Compared with the cumulative number of reported cases in China and Italy, as well as the capacity to provide medical resources, i.e. the number of beds, it is clear that there is a run on medical resources or a shortage of medical resources in Italy, which is much more serious than that in China. Spain and Iran are the slowest in terms of capacity to supplement medical resources, which may also be one of the reasons for the high cumulative number of reported deaths in the two countries. It is also closely related to the recovery rate discussed below.

**Recovery rate and disease-induced death rate of isolated cases in hospital**$\gamma_{2}$ and $\alpha_{2}$

As for the recovery and mortality rates, since the epidemic data only report the number of confirmed or in-hospital cases, we can only compare the recovery and disease-induced death rates of isolated in-hospital patients here. The hospital treatment time in South Korea, Spain, Italy and China is relatively short. The mortality rate of patients in hospital is the highest in Spain, Iran and Italy, and the lowest in China. The reason for the high mortality rate in Japan may be that only severely ill patients are admitted to hospitals there.

**SI4. The data and uncertainty analyses**

We obtained the numbers of daily confirmed cases, cumulative numbers of deaths and other data on COVID-19 in mainland China from NHCC [6]; and those in South Korea, Italy, Japan, Spain and Iran from the KCDC[7], MSPC[8] and WHO[9], respectively. The critical times for strengthening or changing national prevention and control measures can be found in publications [3-9].

In order to analyze the influence of data randomness on parameter estimation and model prediction, we assumed that the epidemic data of each country follows a Poisson distribution, and we randomly generated 1000 columns of datasets for fitting. We then obtained the 95% confidence intervals for the curves generated by the real data estimations in Figs. 2 and 3.

**References**

1. Tang B, Xia F, Tang S, et al. The effectiveness of quarantine and isolation determine the trend of the COVID-19epidemics in the final phase of the current outbreak in China. Int. J. Inf. Dis. 2020;96(2020):636-647.
2. Tang SY, Tang B, Bragazzi NL, et al. Analysis of COVID-19 epidemic traced data and stochastic discrete transmission dynamic model (in Chinese). Sci Sin Math. 2020; 50: 1-16.
3. World Health Organization (WHO). Statement on the meeting of the International Health Regulations (2005) Emergency Committee regarding the outbreak of novel coronavirus (2019-nCoV).2020; https://www.who.int/news-room/detail/23-01-2020-statement-on-the-meeting-of-the-international-health-regulations-(2005)-emergency-committee-regarding-the-outbreak-of-novel-coronavirus-(2019-ncov).
4. Wikipedia. https://en.wikipedia.org/wiki/2020_coronavirus_pandemic_in_Iran.
5. Wikipedia. <https://en.wikipedia.org/wiki/2020_coronavirus_pandemic_in_Spain>.
6. NHCC: National Health Commission of the People’s Republic of China. http://www.nhc.gov.cn/xcs/yqtb/202003/097e6e91ecb6464ea69fd1a324c9b1b4.shtml.
7. KCDC: Korea Centers for Disease Control and Prevention. https://www.cdc.go.kr/board/board.es?mid=a30402000000&bid=0030.
8. MSPC: Ministero della Salute, Protezione Civile. http://www.salute.gov.it/portale/nuovocoronavirus/homeNuovoCoronavirus.jsp.
9. WHO: World Health Organization. Available from: https://www.who.int/emergencies/diseases/novel-coronavirus-2019/situation-reports
